# Surgical treatment of multiple rib fractures and flail chest in trauma: a one-year follow-up study

**DOI:** 10.1186/s13017-016-0085-2

**Published:** 2016-06-14

**Authors:** Eva-Corina Caragounis, Monika Fagevik Olsén, David Pazooki, Hans Granhed

**Affiliations:** Department of Surgery, Institute of Clinical Sciences, Sahlgrenska Academy, University of Gothenburg, Gothenburg, Sweden; Department of Physical Therapy, Institute of Neuroscience and Physiology, Sahlgrenska Academy, University of Gothenburg, Gothenburg, Sweden

**Keywords:** Trauma, Rib fractures, Flail chest, Surgery, Lung function, Quality of life

## Abstract

**Background:**

Multiple rib fractures and unstable thoracic cage injuries are common in blunt trauma. Surgical management of rib fractures has received increasing attention in recent years and the aim of this 1-year, prospective study was to assess the long-term effects of surgery.

**Methods:**

Fifty-four trauma patients with median Injury Severity Score 20 (9–66) and median New Injury Severity Score 34 (16–66) who presented with multiple rib fractures and flail chest, and underwent surgical stabilization with plate fixation were recruited. Patients responded to a standardized questionnaire concerning pain, local discomfort, breathlessness and use of analgesics and health-related quality of life (EQ-5D-3 L) questionnaire at 6 weeks, 3 months, 6 months and 1 year. Lung function, breathing movements, range of motion and physical function were measured at 3 months, 6 months and 1 year.

**Results:**

Symptoms associated with pain, breathlessness and use of analgesics significantly decreased from 6 weeks to 1 year following surgery. After 1 year, 13 % of patients complained of pain at rest, 47 % had local discomfort and 9 % used analgesics. The EQ-5D-3 L index increased from 0.78 to 0.93 and perceived overall health state increased from 60 to 90 % (*p* < 0.0001) after 6 weeks to 1 year. Lung function improved significantly with predicted Forced vital capacity and Peak expiratory flow increasing from 86 to 106 % (*p* = 0.0002) and 81 to 110 % (*p* < 0.0001), respectively, from 3 months to 1 year after surgery. Breathing movements and range of motion tended to improve over time. Physical function improved significantly over time and the median Disability rating index was 0 after 1 year.

**Conclusions:**

Patients with multiple rib fractures and flail chest show a gradual improvement in symptoms associated with pain, quality of life, mobility, disability and lung function over 1 year post surgery. Therefore, the final outcome of surgery cannot be assessed before 1 year post-operatively.

## Background

Multiple rib fractures occur in 10 % of poly-traumatized patients due to blunt, high-energy trauma [1] and can lead to unstable thoracic cage injuries or flail chest [2] with respiratory insufficiency. Whilst conservative management with analgesics and ventilator support has been the conventional treatment for flail chest, this can entail long hospitalization with immobilization, which leads to complications, such as pulmonary infections and long-term disability with chronic pain [3, 4]. Recently, a number of new fixation devices and better techniques have been developed for surgical treatment of rib fractures [5]. Three small, prospective, Randomized Controlled Trials (RCTs) suggest that surgical management of flail chest may decrease the need for ventilator support and intensive care [6–8]. In our clinical setting, we have found plate fixation of rib fractures to be a safe method with a low rate of complications and a reduced time and need for ventilator treatment in comparison to conservatively-managed, historical controls [9]. Our patients were reported to have experienced mild disability, decreased range of motion and lung function, and 35 % of patients had enduring pain after 6 months [10]. Long-term studies concerning lung function, mobility, pain and Quality of Life (QoL) after surgery are lacking. Two studies have reported significantly better lung function in surgically-managed patients 1 month after surgery [6, 7], whereas Marasco et al*.* [8] found no significant difference between operated and conservatively-managed patients after 3 months. There is a disparity in the surgical techniques used in these studies, which makes comparison difficult.

The aim of this prospective study was to examine the long-term patient outcomes associated with pain, physical function, QoL and lung function after surgical stabilization of multiple rib fractures or flail chest.

## Methods

A consecutive series of 60 patients who underwent surgical fixation of multiple rib fractures as a result of blunt trauma were included in a prospective study during the period 2010–2013 [9]. The inclusion criteria for this study were: (i) Flail chest defined as three or more adjacent ribs each fractured in more than one location [2], with respiratory insufficiency (ii) Multiple rib fractures (>4) with respiratory insufficiency and also in need of a thoracotomy due to bleeding or air leakage. Respiratory insufficiency was defined as failing arterial oxygenation despite oxygen administration. Additional information on the 60 patients can be found in a previously published feasibility study [9]. Patients with severe head injury and spinal cord injury were excluded from this follow-up study.

Pre-operative Three-Dimensional (3D) reconstructions of Computer Tomography (CT) images of the thorax were used for planning the surgical procedure. The 3D reconstructions were based on images with 0.625 mm slice thickness and produced in the program AW Volume Share™ 5 (GE Healthcare). Patients were intubated with a double lumen endotracheal tube. A non-muscle sparing thoracotomy was performed to clean out hematoma and debris, identify and, if necessary, manage intra-thoracic injuries. The reason for a non-muscle sparing approach was to gain good access to the chest wall and the multiple fractured ribs. The MatrixRIB**®** (DePuy Synthes) Fixation System consisting of pre-shaped angular locked plates in titanium and intra-medullary splints was used to stabilize rib fractures. Post-operative pain was managed using either an intra-pleural or epidural catheter with adjunct, oral pain medication. Intravenous broad-spectrum antibiotic therapy was given prophylactically until the chest tubes had been removed. Low-molecular weight heparin was given subcutaneously as thrombotic prophylaxis. A surgeon assessed patients at 6 weeks, 3 months, 6 months and 1 year post-operatively using a standardized questionnaire concerning pain, local discomfort, breathlessness and analgesics and QoL according to EQ-5D-3 L [11]. Pain was defined as a strong distressing sensation whereas local discomfort was defined as an unpleasant or abnormal sense to touch. The EQ-5D-3 L results were converted to a single summary index using the Time Trade-Off (TTO) technique with a Swedish reference value set [12]. A chest X-ray was taken 6 weeks post-operatively to assess the presence of lung disease and implant dysfunction or migration. A physiotherapist assessed a subgroup of patients (*n* = 16) at 3 months, 6 months and 1 year post-operatively. The selected patients all had flail chest, no co-morbidities and spoke Swedish. Standardized lung function tests [13] were performed and Forced Vital Capacity (FVC), Forced Expiratory Volume in one second (FEV1) and Peak Expiratory Flow (PEF) were recorded using an EasyOne**®** Spirometer (ndd Medical Technologies Inc., MA, Us). Breathing movements were measured at rest and during maximal breathing by using a Respiratory Movement Measuring Instrument, RMMI**®** (ReMo Inc. Keldnaholt, Reykjavik, Iceland) [14]. The range of motion in the thorax was assessed by measuring thoracic excursion (at the level of the 4^th^ costae and the xiphoid process), flexion and lateral flexion in a standardized manner [10]. Physical function was estimated by using the Disability Rating Index (DRI) questionnaire [15] where 100 is the worst possible outcome and 0 is the best.

The SAS® statistical software package (NC, USA) was used for all statistical analyses. Results are presented as median with range or mean with standard deviation (SD) for continuous variables and n and % for categorical variables. For comparison over time, the Wilcoxon Signed Rank test was used for continuous variables and Sign test was used for categorical variables. The significance level was considered *p* < 0.05.

## Results

**Fig. 1:**
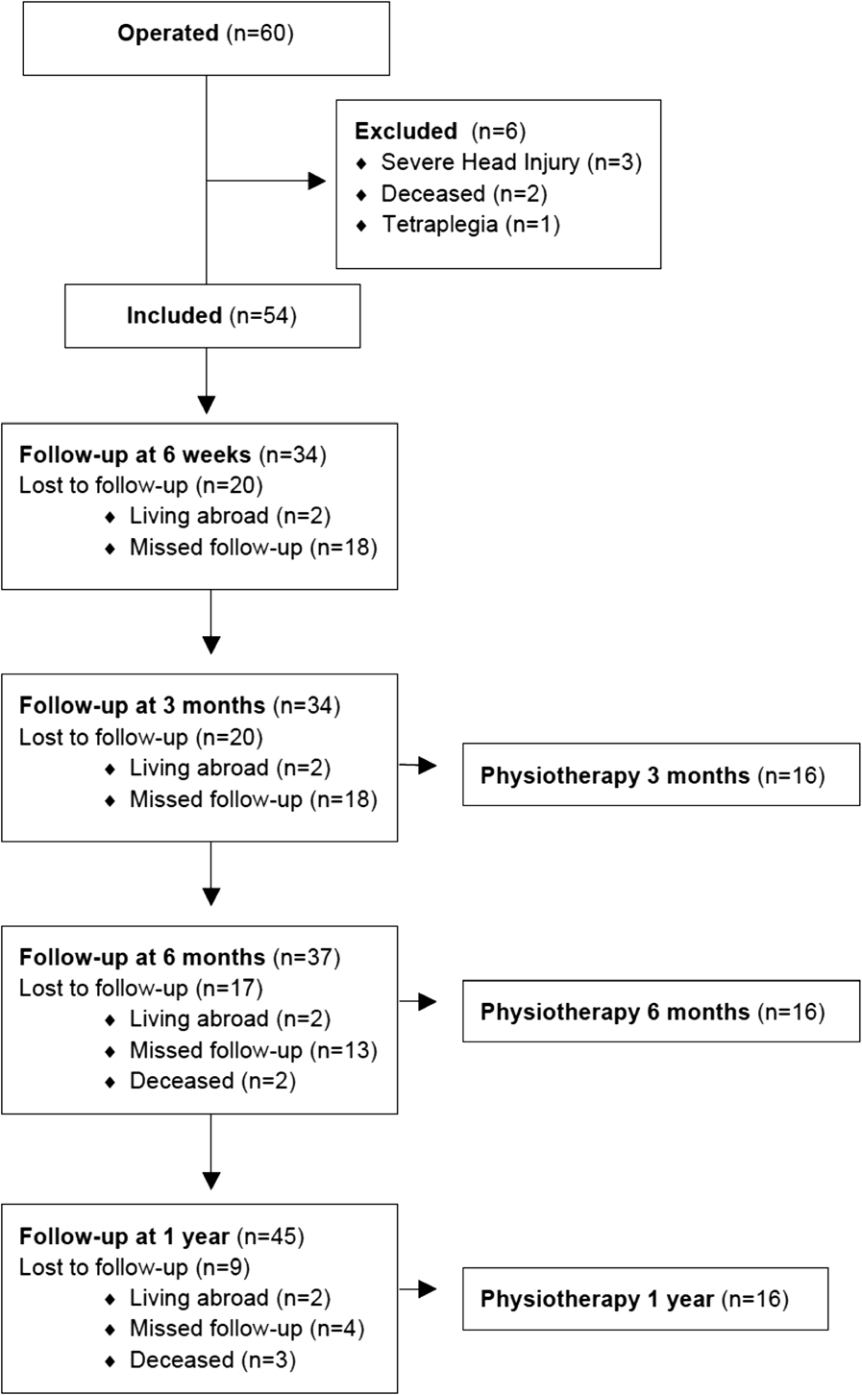
Flow-chart of patients included in the study

Of the 60 patients operated six were excluded from this follow-up. Two patients died during the immediate post-operative period due to multiple organ system failure and respiratory insufficiency. Four patients were excluded due to concomitant injuries resulting in tetraplegia in one case and severe head injury in three patients. Of the 54 patients included in the study 49 patients participated while 5 patients were lost to follow-up (Fig. 1). Nineteen patients were on ventilator pre-operatively and 24 patients were on ventilator 24 h post-operatively. The mechanism of injury was in 92 % of cases either traffic accidents (59 %) or falls (33 %). The indication for surgery was flail chest in 51 patients, bleeding in two patients and air leakage in one patient. The included patients consisted of 40 (74 %) men and 14 (26 %) women with the median age of 57 years (20–86), median Injury Severity Score (ISS) 20 (9–66) and median New Injury Severity Score (NISS) 34 (16–66). Between 63–83 % of included patients in our study attended each follow-up but only 22 patients attended all dates. A physiotherapist assessed 16 patients; ten men and six women, at 3 months, 6 months and 1 year post-operatively. There was a larger proportion of women in the subgroup assessed by a physiotherapist, otherwise age, ISS and NISS was comparable to the overall study group.

The proportion of patients seen at follow-up with pain at rest decreased with 26 % (*p* = 0.039) between 6 weeks and 3 months after surgery. Problems associated with pain on breathing and breathlessness decreased progressively by 21 % (*p* = 0.039) and 27 % (*p* = 0.022), respectively, during the first post-operative year. After 1 year, 13 % of patients complained of pain at rest, 9 % experienced pain on breathing and 16 % experienced breathlessness. Local discomfort did not decrease with time, but remained constant and affected approximately 47 % of patients. Despite enduring pain, there was a significant, 49 % (*p* < 0.0001) decrease in the proportion of patients using analgesia, with 9 % on pain medication after 1 year (Table 1).

**Table 1:** Proportion of patients at follow-up with subjective symptoms after rib fracture surgery

Symptoms	6 weeks (*n* = 34)	3 months (*n* = 34)	6 months (*n* = 37)	1 year (*n* = 45)
Pain at Rest	12 (35.3 %)	4^a^* (11.8 %)	6 (16.2 %)	6^c^* (13.3 %)
Pain on Breathing	8 (23.5 %)	5 (14.7 %)	3 (8.1 %)	4^c^* (8.9 %)
Local Discomfort	14 (41.2 %)	17 (50.0 %)	19 (51.4 %)	21 (46.7 %)
Breathlessness	14 (41.2 %)	12 (35.3 %)	10 (27.0 %)	7^c^* (15.6 %)
Analgesia Usage	18 (52.9 %)	13 (38.2 %)	5^b^** (13.5 %)	4^c^*** (8.9 %)

* *p*-value <0.05

** *p*-value <0.01

*** *p*-value <0.001

^a^ Difference from 6 weeks to 3 months (*n* = 27)

^b^ Difference from 6 weeks to 6 months (*n* = 27)

^c^ Difference from 6 weeks to 1 year (*n* = 33)

**Fig. 2:**
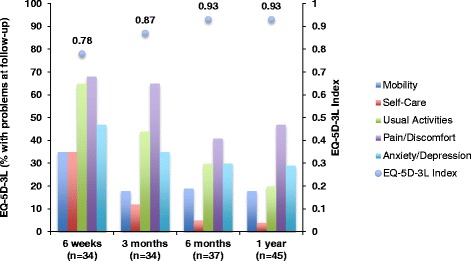
Quality of life measured by EQ-5D-3 L, showing the percentage of patients with some or extreme difficulties and median EQ-5D-3 L index values at 6 weeks, 3 months, 6 months and 1 year follow-up

Patients’ QoL measured with EQ-5D-3 L showed median index values that progressively increased from 0.78 at 6 weeks to 0.93 after 1 year, with the greatest improvement occurring between 6 weeks and 3 months after surgery (Fig. 2). There was a significant decrease in the proportion of patients experiencing problems with mobility (27 %, *p* = 0.022), self-care (36 %, *p* = 0.0005), performance of usual activities (55 %, *p* = 0.0001), and pain or discomfort (27 %, *p* = 0.035) from 6 weeks to 1 year after surgery. There was no significant improvement in symptoms of anxiety or depression over time. The QoL measured by Visual Analogue Scale (VAS) improved significantly over time: median VAS was 60 % (20–96) at 6 weeks, 76 % (40–97) at 3 months, 80 % (20–100) at 6 months and 90 % (30–100) after 1 year. Quality of life significantly increased (30 %, *p* < 0.0001) between 6 weeks to 1 year following surgery.

Percent predicted FVC improved by 6.88 % (SD 5.84; *p* = 0.0002) from 3 to 6 months and by 19.8 % (SD 14.1; *p* = 0.0002) from 3 months to 1 year after surgery. There was no significant improvement in FEV1 over time. There was no significant improvement in mean predicted PEF between 3 and 6 months, but PEF significantly increased by 28.5 % (SD 20.4; *p* < 0.0001) from 3 months to 1 year. After 1 year the mean FVC was 106 %, PEF was 110 % and FEV1 was 80 % compared to predicted values (Table 2).

**Table 2:** Lung function of patients with flail chest (*n* = 16) at 3, 6 and 12 months after surgery

Lung Function	3 months % Predicted	6 months % Predicted	Δ 3–6 months % Predicted	Δ 3–6 months *P*-value	12 months % Predicted	Δ 3–12 months % Predicted	Δ 3–12 months *P*-value
FVC (L)	86.2 ± 19.4	93.1 ± 20.7	6.9 ± 5.8	*p* = 0.0002	105.9 ± 17.5	19.8 ± 14.1	*p* = 0.0002
FEV1 (L)	79.4 ± 22.7	81.8 ± 25.3	2.3 ± 7.5	*p* = 0.100	80.4 ± 29.6	0.95 ± 31.9	*p* = 0.74
PEF (L/min)	81.4 ± 19.5	83.7 ± 24.3	2.3 ± 16.5	*p* = 0.50	109.9 ± 24.8	28.5 ± 20.4	*p* < 0.0001

Breathing movements were decreased on the operated side of the thorax as compared to the non-operated side 3 months after surgery. The operated side showed a tendency towards improved movement over time and better results at 1-year follow-up as regards movements of the upper thorax at rest as compared to the non-operated side (Table 3). Ranges of motion at different levels in the thorax were measured at 3 months, 6 months and 1 year after surgery. Lower level thoracic excursion improved significantly between 3 and 6 months, thoracic flexion and thoracic extension improved significantly between 3 months and 1 year, otherwise movement stayed unchanged (Table 3). Physical function increased significantly over time (Table 3). Between 3 and 6 months, there was an 8.7 mm (*p* = 0.047) decrease in median DRI and between 3 months and 1 year, there was an 18.4 mm (*p* = 0.013) decrease with a median DRI of 0. Of the subset of 16 patients, the number who experienced no disability was three at 3 months, six at 6 months and 10 after 1 year (Table 3).

**Table 3:** Breathing movements, range of motion and disability rating index (DRI) of patients with flail chest (*n* = 16) 3, 6 and 12 months after surgery

Breathing Movements (*n* = 16)	3 months Mean ∆ Operated vs Non-Operated side	6 months Mean ∆ Operated vs Non-Operated side	1 year Mean ∆ Operated vs Non-Operated side
Rest			
Upper Thorax (mm)	-0.76 ± 1.12	-0.40 ± 0.68	0.49 ± 1.32^b^**
Lower Thorax (mm)	-0.22 ± 0.88	0.10 ± 0.56	0.27 ± 0.82
Abdominal (mm)	-0.20 ± 1.67	0.43 ± 1.63	0.14 ± 1.40
Maximal Breathing			
Upper Thorax (mm)	-3.04 ± 5.24	-1.24 ± 1.77	0.10 ± 4.88
Lower Thorax (mm)	-0.05 ± 4.57	1.48 ± 3.62	1.05 ± 4.46
Abdominal (mm)	-0.91 ± 4.92	-0.58 ± 2.64	0.65 ± 5.09
Range of Motion (*n* = 16)	3 months Mean	6 months Mean	1 year Mean
Upper level Thoracic Excursion (cm)	3.84 ± 1.71	4.09 ± 1.53	3.98 ± 1.58
Lower level Thoracic Excursion (cm)	3.41 ± 1.29	4.38 ± 1.70^a^*	3.82 ± 1.61
Thoracic Flexion (cm)	1.75 ± 0.88	2.06 ± 1.03	2.25 ± 0.75^b^*
Thoracic Extension (cm)	0.66 ± 0.47	0.88 ± 0.43	1.17 ± 0.45^b^**
Lateral Flexion towards injured side (cm)	14.50 ± 3.80	15.50 ± 5.30	14.10 ± 6.20
Lateral Flexion from injured side (cm)	14.80 ± 5.40	15.90 ± 4.20	14.40 ± 6.40
DRI (*n* = 16)	3 months Median	6 months Median	1 year Median
(0-100 mm)	23.0 (0.0–78.1)	15.3^a^* (0.0–65.3)	0.0^b^* (0.0–66.9)

* *p*-value <0.05

** *p*-value <0.01

^a^ Difference from 3 months to 6 months

^b^ Difference from 3 months to 1 year

One patient developed osteomyelitis and underwent a re-operation with plate extraction after 7 months with cessation of infection as a result. A second re-operation was performed on a professional athlete who experienced local pain at the site of a protruding plate when practicing his sport. One patient had a loose screw on the plate, whilst another patient had a loose intra-medullary splint on chest X-rays 6 weeks post-operatively; however neither patient experienced clinical symptoms and they were not re-operated. Three patients (5.6 %) died within 1 year after surgery; one patient died after 65 days due to cardiac arrest, one patient died after 137 days due to complications of Chronic Obstructive Lung Disease (COPD) and the third patient died after 277 days due to osteomyelitis in the tenth thoracic vertebra.

## Discussion

In this prospective study of 54 trauma patients who underwent stabilizing surgery of rib fractures, we found progressive improvement in pain, mobility, activity, QoL, lung function and disability during the first, post-operative year.

The primary end-point in previous studies of surgical treatment of flail chest has mainly focused on aspects associated with respiratory insufficiency. Although acute pain can contribute to respiratory problems, chronic pain can be debilitating and lead to decreased QoL. We found that 13 % of our patients experienced enduring chest pain at rest after 1 year. In contrast, a previous observational study of conservatively-managed patients with flail chest showed that 49 % experienced enduring pain after a mean follow-up of 5 years [4]. It is probable that surgery decreases chronic pain. In the prospective RCT of Tanaka et al., symptoms of chest tightness, thoracic cage pain and dyspnea on effort were more frequent in conservatively-managed patients [6]. Surgery *per se* is associated with morbidity, however, and 47 % of our patients experienced some form of local discomfort, although it was unclear whether this was due to the trauma or surgery. Despite enduring pain and discomfort, only 9 % of patients used analgesics, suggesting mild and not particularly disabling symptoms. Patient QoL improved gradually after surgery; the median EQ-5D-3 L VAS was 90 % after 1 year, which is higher than that of a Swedish population study, which showed a mean VAS of 77.4 and 75.8 % for men and women, respectively [16]. The median EQ-5D-3 L index was 0.93 in our patients 1 year after surgery, which is higher than that of healthy individuals in the population study mentioned [16], where men had 0.84 and women 0.80. We found that our patients did not improve significantly in the dimension concerning anxiety or depression. It is possible that these were pre-existing problems since trauma patients are often afflicted with psychiatric problems and substance abuse [17], but it is also probable that the trauma itself is a cause of persisting anxiety and depression. The only RCT to compare QoL in operated and conservatively managed patients found no difference between the groups at 6 months follow-up [8].

Lung function in a subgroup of patients improved significantly over time and patients reached FVC and PEF values of greater than 100 % of predicted values after 1 year. The FEV did not improve over time and was approximately 80 % of the predicted value, which indicates a remaining obstructive component. Tanaka et al*.* [6] found progressive improvement in FVC during a follow-up period of 1 year, where patients stabilized with Judet struts had consistently significantly better results compared to conservatively-managed patients. These patients, however, did not reach the 100 % and above values in predicted lung function, as seen in our study. Granetzny et al*.* [7] used Kirschner wires to stabilize patients and also showed better lung function compared to conservatively-managed patients, 2 months after surgery, whereas Marasco et al*.* used biodegradable inion plates and found no difference in lung function after 3 months [8]. Whilst it takes the inion plate 18–24 months to resorb, it gradually loses most of its strength within 18–36 weeks and might weaken before the bone is fully healed. Considering the very different approaches to surgical stabilization in the aforementioned studies, comparison of results is difficult. Based on our studies [9, 10], we believe that surgical stabilization using the MatrixRIB**®** (DePuy Synthes) Fixation System is superior since the plates mimic the biodynamic characteristics of the ribs and are fixed with angular locked screws creating stability in movement without losing strength over time.

None of the RCTs have studied breathing movements and range of motion at follow-up. We found that breathing movements were decreased on the operated side, but tended to improve gradually over time, and whilst a statistically significant improvement was found in movements of the upper thorax at rest, these changes were not clinically significant and there was no major difference between operated and non-operated side. The range of motion was statistically improved as regards the lower level thoracic excursion, flexion and extension but the changes were small and of little clinical relevance. Physical function, assessed by DRI, improved gradually and significantly with time; the median was 0 after 1 year, indicating no loss of function. However, the range of 0–67 suggests a spread, with some patients experiencing some difficulty (≥25) or difficulty (≥50) in function. The results may also reflect long-term outcome of concomitant injuries; however, we found that at least 50 % of trauma patients who have undergone surgical fixation of flail chest have no remaining disability after 1 year. Previous studies of conservatively-managed patients with flail chest have shown that 66 and 38 % of them experienced persistent disability after 2 months [3] and during a mean follow-up period of 5 years (6 months-12 years) [4], respectively. Marasco et al*.* found that 71 % of conservatively managed patients experienced daily limitations and disabilities after 3 months compared to 48 % of operated patients, suggesting that surgical management of flail chest decreases prolonged disability, although this may be particularly evident in cases of isolated thoracic injuries.

Late complications were seen in four out of 60 patients with two undergoing re-operations due to deep wound infection and subjective symptoms from a protruding plate. We consider this a low rate of complications considering these were among the first patients operated at our centre.

The results of this study are limited as this was an uncontrolled study. The patients included were mostly subjected to poly-trauma, as demonstrated by a median ISS of 20. Poly-trauma patients are an inherent, heterogeneous group with associated injuries in addition to their thoracic trauma, which serve as confounding factors influencing the perception of pain, function, activity and QoL. Results from patients with isolated thoracic injury are likely to have been more homogenous and easier to interpret, but such a group would not have been representative of the population at large. Even in isolated thoracic injury, however, there are confounding factors due to commonly associated clavicle and scapular fractures that influence the mobility, function and pain of the thoracic cage. Moreover, the pre-existing pain, function and disability were unknown for patients in this study. Between 63–83 % of patients in our study attended each follow-up but only 22 patients attended all dates, which poses a weakness and a potential bias in the results. However, the age, sex, ISS and NISS values compared between included patients and those at each follow-up were similar.

Surgical treatment of rib fractures has received increasing attention in recent years. While previous studies have largely focused on the treatment of flail chest, it is not clearly defined if patients with multiple rib fractures or dislocated ribs also benefit from surgery. We chose to study the long-term results of patients with flail chest as this group has been the main focus of previous studies. However, patients with multiple rib fractures that required surgery for other reasons, such as air leakage or bleeding, were also stabilized and therefore included in this study. With the development of minimally invasive approaches to rib fixation more will be gained from surgery and the indications may well include multiple rib fractures in the future. A muscle-sparing approach to the ribs and a selective usage of thoracotomy would presumably decrease post-operative pain. However, muscle-sparing techniques minimize access to the injured chest wall making it difficult to fixate multiple rib fractures. Video-assisted thoracoscopic surgery (VATS) could be used to clear out hematoma and resect leaking lung tissue. However, the technique does require lung deflation to some extent, which may not be possible in all trauma patients with severe lung contusions.

The method used for stabilizing ribs varies between different studies, making comparison difficult as the biomechanical properties of the implants differ. There is a need for larger prospective RCTs that compare not only the outcome of surgery concerning ventilator support and Intensive Care Unit (ICU) care, but also the long-term outcomes associated with pain, function, activity and QoL, as well as cost-benefit of such surgical management.

## Conclusions

Patients who underwent surgical plate fixation of multiple rib fractures and flail chest showed a gradual improvement in symptoms associated with pain, physical function, lung function and QoL, which continued throughout the first post-operative year. Breathing movements, range of motion, symptoms of anxiety or depression, and local discomfort did not improve significantly over time. We conclude that the final outcome after plate fixation of rib fractures cannot be assessed before 1 year post-operatively.

## Abbreviations

3D, Three-Dimensional; COPD, Chronic Obstructive Lung Disease; CT, Computer Tomography; DRI, Disability Rating Index; FEV1, Forced Expiratory Volume in One second; FVC, Forced Vital Capacity; ICU, Intensive Care Unit; ISS, Injury Severity Score; NISS, New Injury Severity Score; PEF, Peak Expiratory Flow; QoL, Quality of Life; RCT, Randomized Controlled Trial; RMMI, Respiratory Movement Measuring Instrument; SD, Standard Deviation; TTO, Time Trade-Off; VAS, Visual Analogue Scale.
